# Association between stroke occurrence and changes in atmospheric circulation

**DOI:** 10.1186/s12889-020-10052-5

**Published:** 2021-01-06

**Authors:** Jone Vencloviene, Ricardas Radisauskas, Daina Kranciukaite-Butylkiniene, Abdonas Tamosiunas, Vidmantas Vaiciulis, Daiva Rastenyte

**Affiliations:** 1 Department of Environmental Sciences, Vytautas Magnus University, Donelaicio St. 58, LT-44248 Kaunas, Lithuania; 2 Institute of Cardiology, Lithuanian University of Health Sciences, Sukileliu St. 15, LT-50103 Kaunas, Lithuania; 3 Department of Environmental and Occupational Medicine, Lithuanian University of Health Sciences, Tilzes St. 18, LT-47181 Kaunas, Lithuania; 4 Department of Family Medicine, Lithuanian University of Health Sciences, Eiveniu St. 2, LT-50009 Kaunas, Lithuania; 5 Department of Preventive Medicine, Lithuanian University of Health Sciences, Tilzes St. 18, LT-47181 Kaunas, Lithuania; 6 Health Research Institute, Lithuanian University of Health Sciences, Tilzes St. 18, LT-47181 Kaunas, Lithuania; 7 Department of Neurology, Lithuanian University of Health Sciences, Eiveniu St. 2, LT-50009 Kaunas, Lithuania

**Keywords:** Ischaemic stroke, Haemorrhagic stroke, Atmospheric circulation, Arctic oscillation, North Atlantic oscillation, East Atlantic/West Russia indices, El Niño-southern oscillation

## Abstract

**Background:**

The impact of weather on morbidity from stroke has been analysed in previous studies. As the risk of stroke was mostly associated with changing weather, the changes in the daily stroke occurrence may be associated with changes in atmospheric circulation. The aim of our study was to detect and evaluate the association between daily numbers of ischaemic strokes (ISs) and haemorrhagic strokes (HSs) and the teleconnection pattern.

**Methods:**

The study was performed in Kaunas, Lithuania, from 2000 to 2010. The daily numbers of ISs, subarachnoid haemorrhages (SAHs), and intracerebral haemorrhages (ICHs) were obtained from the Kaunas Stroke Register. We evaluated the association between these types of stroke and the teleconnection pattern by applying Poisson regression and adjusting for the linear trend, month, and other weather variables.

**Results:**

During the study period, we analysed 4038 cases (2226 men and 1812 women) of stroke. Of these, 3245 (80.4%) cases were ISs, 533 (13.2%) cases were ICHs, and 260 (6.4%) cases were SAHs. An increased risk of SAH was associated with a change in mean daily atmospheric pressure over 3.9 hPa (RR = 1.49, 95% CI 1.14–1.96), and a stronger El Niño event had a protective effect against SAHs (RR = 0.34, 95% CI 0.16–0.69). The risk of HS was positively associated with East Atlantic/West Russia indices (RR = 1.13, 95% CI 1.04–1.23). The risk of IS was negatively associated with the Arctic Oscillation index on the same day and on the previous day (RR = 0.97, *p* < 0.033). During November–March, the risk of HS was associated with a positive North Atlantic Oscillation (NAO) (RR = 1.29, 95% CI 1.03–1.62), and the risk of IS was negatively associated with the NAO index (RR = 0.92, 95% CI 0.85–0.99).

**Conclusions:**

The results of our study provide new evidence that the North Atlantic Oscillation, Arctic Oscillation, East Atlantic/West Russia, and El Niño-Southern Oscillation pattern may affect the risk of stroke. The impact of these teleconnections is not identical for various types of stroke. Emergency services should be aware that specific weather conditions are more likely to prompt calls for more severe strokes.

## Background

During the past decade, many studies have focussed on the impact of weather on morbidity from stroke, and several meta-analyses or reviews have been carried out [1**–**5]. Air temperature (T) and atmospheric pressure (AP) are among the most closely studied weather variables; increases, decreases, and fluctuations in both have been significantly linked to numerous stroke subtypes. A statistically significant negative association has been observed between T and the incidence of ischaemic stroke (IS) [6**–**8], haemorrhagic stroke (HS) [9, 10], intracerebral haemorrhage [11**–**14]], and subarachnoid haemorrhage [15**–**17]. The results of a meta-analysis [4] have also shown a negative association between daily T and all stroke subtypes. The associations between stroke incidence and T were stronger in Europe and were not significant in North America [4]. Daily T changes have also been associated with the risk of stroke. The results of a meta-analysis have shown that a daily increase in T was a protective factor against HS, and an increase as well as a decrease in daily T acted as a risk factor for IS [1].

Studies have shown both a negative association [1, 12, 18] and a positive association [14, 15, 19] between the risk of stroke and AP. Studies conducted predominantly in Europe have shown an increase in the incidence of stroke associated with AP changes, adjusting for T or seasonality [19**–**22]. According to a study conducted in Germany, rapid decreases in ambient temperature and rapid changes in relative humidity and AP increase the risk of IS under temperate climate conditions [23]. These results showed that the risk of stroke occurrence was associated with changing weather. It is possible that the risk of stroke occurrence was associated with some patterns of atmospheric variability.

The changes in atmospheric circulation are regulated through teleconnections – large-scale patterns of pressure and circulation anomalies that cover vast geographic areas. These patterns reflect large-scale changes in atmospheric waves and influence T, rainfall, storm tract, and jet streams [24]. The most important teleconnection pattern in the Atlantic-European region is the North Atlantic Oscillation (NAO), characterised as a dipole in the sea-level pressure between the Azores high and the Icelandic low [25]. Other teleconnections such as the Arctic oscillation (AO), Scandinavian pattern (SCA), and East Atlantic/West Russia pattern (EA/WR) also determine weather variability in the Northern Hemisphere [26**–**27]. Several studies have also suggested a consistent and statistically significant impact of the El Niño-Southern Oscillation (ENSO) over the North Atlantic and Europe [28].

The NAO is the most prominent and recurrent pattern of atmospheric variability over the middle and high latitudes of the Northern Hemisphere, especially during the cold season months (November–March) [25]. During winter, in Northern Europe, the positive NAO phase was associated with a stronger westerly wind flow, a higher T, and increased storminess and precipitation, whereas the negative NAO phase led to a weakened westerly wind, a lower temperature, and decreased storminess and precipitation [25]. Apart from the NAO, other teleconnections such as AO, SCA, EA/WR, and ENSO also determine climate variability in the Northern Hemisphere and regulate the frequency and intensity of significant weather events [29–33].

As the risk of stroke has been associated mostly with changing weather, the changes in the daily stroke occurrence are probably associated with changes in atmospheric circulation. The teleconnections affecting the variability in atmospheric circulation in the Baltic region are likely to determine certain weather patterns or events that are potentially associated with the risk of stroke. The aim of this study was to detect the complex association between the daily numbers of ISs and HSs in patients aged 25–64 years and teleconnection indices – daily NAO, AO, and ENSO indices and monthly indices of EA/WR and SCA, adjusting for weather variables. In our previous study [34], the impact of T, AP, relative humidity (RH), and wind speed (WS) on the risk of stroke was evaluated. As over the middle and high latitudes of the Northern Hemisphere, the most prominent and recurrent pattern of atmospheric variability is the NAO, especially during the cold season months [25], we assessed the associations between NAO indices and the risk of strokes separately during November–March and April–October.

## Methods

### Patients

Data on stroke patients were obtained from the Kaunas population-based Stroke Register database. The registration of stroke cases among middle-aged (25–64 years old) Kaunas residents has been carried out from 1986 to the present day. Data collected for the period of 2000–2010 were used in this study. Stroke registration was conducted according to the WHO MONICA project protocol and established quality control procedures and has been described in detail elsewhere [35, 36]. Multiple sources of information (hospital discharge records, records of outpatient departments, necropsy, medicolegal records, and death certificates) for the stroke event register were used. According to the study protocol, every stroke event must have its apparent onset within the study period and more than 28 days from any previously recorded stroke event in the same case. Multiple stroke attacks occurring within 28 days from onset were considered as a single event. All patients suspected of having died from stroke or having had a nonfatal acute stroke were registered. The codes for the specific types of stroke were confirmed by specific diagnostic examinations. For subarachnoid haemorrhage (SAH) (ICD-10 codes I60.0-I60.9), necropsy (for fatal events), brain computed tomography (CT), or cerebrospinal fluid containing blood was required to determine the diagnosis; for intracerebral haemorrhage (ICH) (ICD-10 codes I61.0-I61.9), the diagnosis had to be confirmed by CT or by necropsy. IS (ICD-10 codes I63.0-I63.9) was diagnosed when CT and/or autopsy could verify the infarction and/or exclude haemorrhage and nonvascular disease.

### Environmental variables

Based on the results of other authors, the weather variables linked to cardiovascular health were used as predictors in the regression model for stroke. In this case, teleconnections associated with changes in the weather pattern of the Baltic region were chosen as predictors. The environmental variables used in our study are routinely collected in certain publicly available databases. The values of daily NAO and AO indices (NAOIs and AOIs) and the monthly EA/WR and SCA indices (EA/WRIs and SCAIs) were obtained from the database of the National Oceanic and Atmospheric Administration (NOAA) (https://www.cpc.ncep.noaa.gov/data/teledoc/telecontents.shtml). We included the daily NINO3.4 index (Equatorial Pacific Sea Surface Temperature), which indicates the effect of ENSO. NINO3.4 indices were taken from the NOAA database (https://climexp.knmi.nl/data/inino34_weekly.dat). Data on the mean daily T (°C), AP (hPa), RH (%), cloud cover (CC) (okta), and WS (m/s) for the studied period were obtained from the Lithuanian Hydrometeorological Service Kaunas Meteorological Station located in the suburbs of Kaunas.

### Statistical analysis

The independence in the daily number of SAHs, ICHs, and ISs was tested by calculating the autocorrelation function. As no significant autocorrelations were detected, the associations between teleconnection indices and the daily number of ISs, SAHs, and ICHs were evaluated by applying a multivariate Poisson regression. First, we assessed the univariate associations between the risk of all types of stroke and weather variables, teleconnection indices, and daily changes (Δ) in T, AP, RH, and WS. As nonlinear and U-shaped associations between weather variables and the risk of acute events were found, the weather variables were used as continuous variables or were categorised. The thresholds of categorical variables were detected by using the classification and regression tree (CRT) method [37]. Apart from this, we analysed the risk of strokes in the tertiles of environmental variables. The weather variables were included one by one in the regression model with predictors: the linear trend, T, and the day of the week and the month (the day of the week and the month were used as categorical variables). In the analysis, we used the environmental variables on the day of the stroke and on the previous 1–2 days (with lags of 0, 1, and 2 days, respectively). The choise of the weather variable (categorical or continuous) and optimal lag was made using the Akaike information criterion. Second, we analysed the impact of teleconnection indices by including the corresponding variables in the multivariate regression model with the linear trend, month, day of the week, T, and other significant weather variables. In the analysis, we used teleconnection indices as continuous and categorised by the tertiles and by using the CRT method. The optimal models were created using the Akaike information criterion. We checked the autocorrelations of the residuals using partial autocorrelation functions for the created model. Third, we analysed the impact of the NAOI on the risk of strokes separately for November–March and April–October. To assess the impact of environmental variables, we presented adjusted rate ratios (RRs) in the multivariate Poisson regression model. Statistical analysis was performed using SPSS 20 software (IBM Corp. in Armonk, NY, USA).

## Results

During the study period, we analysed 4038 cases of stroke (2226 (55.1%) men and 1812 (44.9%) women). Of these, 3245 (80.4%) cases were IS, 533 (13.2%) cases were ICH, and 260 (6.4%) cases were SAH. The analysis of demographic characteristics showed that all types of stroke were more prevalent in the older age group (55–64 years). Two-thirds of all IS occurred in the 55–64 year age group. Both ICH and IS had a higher prevalence among men. The distribution of strokes by sex, age, and stroke type is presented in Table 1.

**Table 1:** Distribution of strokes by sex, age and stroke types

Sex/age groups	Total	SAH	ICH	HS (SAH + ICH)	IS
All, n (%)	4038 (100.0)	260 (6.4)	533 (13.2)	793 (19.6)	3245 (80.4)
Men, n (%)	2226 (55.1)	118 (5.3)	306 (13.7)	424 (19.0)	1802 (81.0)
Women, n (%)	1812 (44.9)	142 (7.9)	227 (12.5)	369 (20.4)	1443 (79.6)
25–54, n (%)	1521 (37.7)	164 (10.8)	224 (14.7)	388 (25.5)	1133 (74.5)
55–64, n (%)	2517 (62.3)	96 (3.8)	309 (12.3)	405 (16.1)	2112 (83.9)

*SAH* subarachnoid haemorrhage, *ICH* intracerebral haemorrhage, *HS* haemorrhage stroke, *IS* ischemic stroke

Over the study period, the mean daily temperature was 7.5 °C, and the daily change in air temperature ranged from − 14.1 to 17.7 °C. The mean daily atmospheric pressure was 1015 hPa, and the daily change in atmospheric pressure ranged from − 36.8 to 32.9. All descriptive characteristics of the environmental variables are presented in Table 2.

**Table 2:** The descriptive characteristics of weather variables

Variable	Mean (SD)	Minimum	Percentiles			Maximum
			25th	50th	75th	
Air temperature, °C	7.5 (8.9)	−2.52	1.0	7.8	14.8	26.6
Daily change in air temperature	0 (2.4)	−14.1	−1.4	0	1.4	17.7
Atmospheric pressure, hPa	1015 (9.6)	976	1009	1015	1021	1050
Daily change in atmospheric pressure	0 (6.0)	−36.8	−3.4	0	3.5	32.9
Relative humidity, %	80.4 (12)	37	73	82	89	100
Daily change in relative humidity	0 (7.8)	−34	−5	0	4	39
Wind speed, m/s	5.3 (2.0)	1	4	5	6	15
Cloud cover, okta	3.5 (1.6)	0.3	2.4	3.3	4.5	11.5
AO index	−0.12 (1.5)	−5.80	−0.95	−0.06	0.79	4.70
NAO index	−0.06 (0.8)	−3.25	−0.58	− 0.02	0.51	2.39
NINO3.4 index	−0.07 (0.8)	−2.20	−0.60	0.00	0.50	1.90
EA/WR indices	−0.17 (0.9)	−2.05	−0.85	− 0.25	0.46	1.79
SCA indices	0.06 (0.9)	−2.33	−0.53	0.00	0.68	2.11

*AO* Arctic Oscillation, *EA/WR* East Atlantic/West Russia, *NAO* North Atlantic Oscillation, *NINO3.4* Equatorial Pacific Sea Surface Temperature, Scandinavian pattern (SCA)

In the multivariate Poisson regression model, a change in daily AP of > 3.9 hPa from the previous day, RH > 96.5% on the previous day, CC > 3.95, and NINO3.4 ≤ 1.14 were statistically significantly associated with an increased rate ratio of SAH (Table 3). In other words, a stronger El Niño (NINO3.4 > 1.14) had a protective impact on SAH (RR = 0.34, 95% CI 0.16–0.69). A dose-response association between NINO3.4 and SAH was found. A change in daily AP of > 9.55 hPa from the previous day with a lag of 1 day, daily change in RH < 7.5%, CC > 1.35 on the previous day, and EA/WRI were associated with an increased RR of ICH. The rate ratio of HS was associated with daily increases in AP and CC > 3.95 and was positively associated with the EA/WR index. In addition, a dose-response association between NINO3.4 and EA/WRI and ICH was detected. The RR of IS was positively associated with an increase in daily T over 2.2 °C compared with the previous day, RH over 53.5% with a lag of 1 day, a strong positive SCAI, NINO3.4 < − 1.60, and a strongly negative EA/WR (EA/WRI < -1.81). Apart from this, the risk of IS was negatively associated with AOI (dose-response association was detected) in the model without other teleconnections but was not statistically significant in the multivariate model (*p* = 0.053) (Table 3).

**Table 3:** Associations^a^ of environmental variables with different types of stroke

Variable	Lag	RR	95% CI	p	RR	95% CI	p
		Univariate association			**Multivariate model**		
**SAH**							
ΔAP (per 10 hPa)	0	1.20	0.98–1.47	0.082			
ΔAP > 3.9 hPa	0	1.48	1.12–1.94	0.005	1.49	1.14–1.96	0.004
RH > 96.5%	1	1.93	1.14–3.28	0.015	2.05	1.21–3.50	0.008
CC > 3.95	0	1.36	1.05–1.76	0.022	1.42	1.09–1.84	0.008
NINO3.4	0	0.87	0.75–1.01	0.065			
NINO3.4 > 1.14	0	0.32	0.16–0.67	0.002	0.34	0.16–0.69	0.003
**ICH**							
ΔT > 2.6 °C	0	1.27	0.98–1.63	0.071			
ΔAP > 9.55 hPa	1	1.48	1.08–2.02	0.014	1.51	1.10–2.06	0.010
RH > 66.5%	1	1.39	1.00–1.93	0.051			
ΔRH < 7.5%	0	1.39	1.06–1.83	0.017	1.42	1.08–1.87	0.013
CC > 1.35	1	2.17	1.16–4.06	0.016	2.17	1.16–4.07	0.016
EA/WRI	0	1.16	1.05–1.30	0.005	1.16	1.04–1.29	0.006
NINO3.4 > 0.11	0	1.23	1.04–1.46	0.017			
**HS**							
ΔAP > 3.9 hPa	0	1.27	1.08–1.49	0.003	1.25	1.06–1.46	0.008
ΔAP > 9.55 hPa	1	1.40	1.07–1.82	0.013	1.36	1.04–1.77	0.023
ΔRH < 7.5%	0	1.32	1.06–1.64	0.015	1.28	1.03–1.46	0.028
CC > 3.95	0	1.20	1.03–1.39	0.017	1.21	1.04–1.41	0.012
EA/WRI	0	1.13	1.04–1.23	0.006	1.13	1.04–1.23	0.006
**IS**							
ΔT > 2.2 °C	0	1.13	1.03–1.25	0.009	1.12	1.01–1.23	0.024
RH > 53.5%	1	1.46	1.12–1.90	0.005	1.41	1.09–1.84	0.010
AOI (per 1)	0	0.97	0.94–0.99	0.008	0.98	0.95–1.00	0.053
AOI (per 1)	1	0.97	0.95–1.00	0.034			
AOI (per 1)	2	0.98	0.96–1.00	0.092			
SCAI	0	1.04	1.00–1.08	0.089			
SCAI> 0.25	0	1.12	1.04–1.21	0.002	1.10	1.02–1.18	0.015
NINO3.4 < −1.60	0	1.25	1.09–1.44	0.002	1.24	1.08–1.43	0.003
EA/WRI < -1.81	0	1.73	1.41–2.12	< 0.001	1.59	1.29–1.96	< 0.001

^a^ adjusting for the linear trend, the month, the day of the week, and air temperature

ΔAP = (mean daily AP on the same day) – (mean daily AP on the previous day)

The effect of EA/WR on the risk of HS was similar in men and women. However, the effects of teleconnection variables on the risk of IS were stronger in women.

**Fig. 1:**
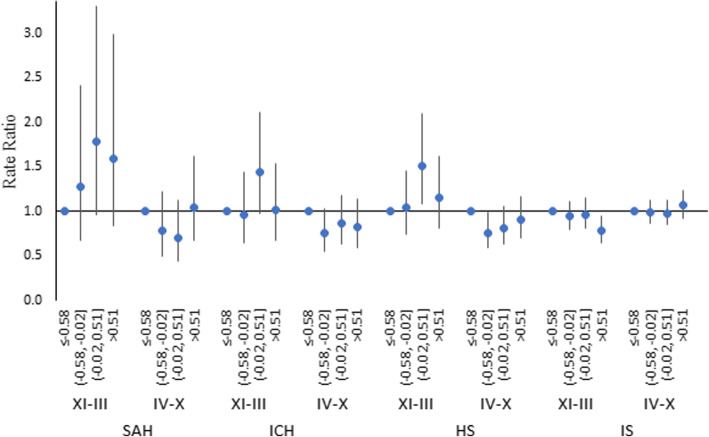
Rate ratios of strokes in the quartiles of the NAOI (reference category – NAOI<-0.58)

No significant associations between the NAOI and the rate ratio of strokes were found throughout the period of the study. The analysis of the RRs of strokes in NAOI quartiles (adjusting for the month, air temperature, and other environmental variables included in the multivariate model (Table 3)) during the period of November–March and April–October showed some impact of NAOI on the risk of stroke (Fig. 1). During November–March, a positive NAO was associated with an increase in the RR of HS, and the RR of IS negatively correlated with the NAOI. During April–October, only NAOI <-0.5 was associated with the RR of HS (Table 4). Moreover, during the colder period, the effect of the NAOI on the risk of IS was stronger than the effect of the AOI. The ENSO variable was negatively correlated with the risk of SAH during November–March and with the risk of IS during April–October (Table 4).

**Table 4:** Associations^a^ of the NAOI with different types of stroke during November–March and April–October

Variable	Lag	RR (95% CI)	P	RR (95% CI)	P
		November–March		**April–October**	
**SAH** NINO3.4	0	0.74 (0.61–0.90)	0.002	1.15 (0.89–1.49)	0.282
NAOI >-0.92	0	2.48 (0.98–56.28)	0.055	0.95 (0.61–1.48)	0.809
**ICH** EA/WRI	0	1.25 (1.05–1.48)	0.013	1.09 (0.95–1.25)	0.209
0 < NAOI < 0.5	0	1.43 (1.08–1.90)	0.012		
NAOI<-0.5	0			1.25 (0.98–1.59)	0.078
**HS** EA/WRI	0	1.14 (0.99–1.32)	0.064	1.10 (0.99–1.23)	0.089
NAOI > 0	0	1.29 (1.03–1.62)	0.030		
NAOI <-0.5	0			1.25 (1.02–1.53)	0.028
**IS (1)** SCAI> 0.25	0	1.08 (0.96–1.21)	0.189	1.12 (1.02–1.24)	0.024
NINO3.4 < −1.60	0			1.54 (1.19–2.00)	0.001
EA/WRI < -1.81	0	1.92 (1.34–2.75)	< 0.001	1.58 (1.22–2.04)	0.001
NAOI (per 1)	0	0.92 (0.85–0.99)	0.028	1.04 (0.98–1.11)	0.166
**IS (2)** SCAI> 0.25	0	1.07 (0.96–1.21)	0.221	1.12 (1.01–1.24)	0.027
NINO3.4 < −1.60	0			1.55 (1.19–2.01)	0.001
EA/WRI < -1.81	0	2.00 (1.40–2.87)	< 0.001	1.59 (1.23–2.05)	< 0.001
NAOI < 0.5	0	1.25 (1.10–1.41)	0.001	0.91 (0.81–1.02)	0.097

^a^ adjusting for the linear trend, the month, the day of the week, and T, RH, and CC variables

## Discussion

In this 11-year study of 260 patients with SAH, 533 patients with ICH, and 3245 patients with IS, we found that some patterns of RH and CC and daily changes in AP and RH were associated with the risk of some types of stroke. In addition, we used the NAO, AO, EA/WR, SCA, and ENSO indices as predictors for the evaluation the risk of stroke. For the first time, we detected a protective effect of warmer ENSO (a stronger El Niño) on the risk of SAH, a positive association between the risk of HS and the EA/WR, a negative association between the rate ratio of IS and the AOI, and a negative impact of a strong positive SCA on the risk of IS. Apart from these results, during November–March, a higher risk of HS was related to a positive NAO, and a negative correlation between the risk of IS and the NAOI was found. In the analysis, an impact of teleconnection indices was detected, adjusting for seasonal variation, T, and other weather variables.

In our study, the risk of HS was associated with daily changes in AP above the threshold of 3.9 hPa for SAH and 9.55 hPa for ICH. These results are in line with those obtained by other authors who found a significant association between SAH and ICH and changing AP. The daily change in AP with a lag of 1 day was positively correlated with the daily number of SAHs in the English Midlands [19], and the daily change in AP > 10 hPa was associated with the risk of SAH in the UK [38] and in Germany [20].

To our knowledge, the risk of SAH was associated with a higher RH and CC level, and the risk of ICH was associated with a higher CC level on the previous day and with a lower daily RH change. Some authors found a significant association between stroke and RH and daily hours of sunshine, which is the opposite variable to cloud cover. A decrease in daily sunlight hours was positively associated with the risk of SAH [17**, **39]. A positive association between SAH and RH was found in the humid subtropical zone [16] and in the Rhein Main area [20], while in the southern regions of France and in the areas with various climatic conditions (41 states of USA), a negative association between SAH and RH was observed [17**,** 39]. ICH is negatively correlated with sunshine hours [40] and positively correlated with the amount of precipitation [6], which coincides with our results.

We found a positive effect of a strong warm ENSO on human health. In our study, most of these events (NINO3.4 > 1.14) fell into colder months. Therefore, the protective effect of a warmer ENSO on SAH may be explained by the effect of ENSO on the weather pattern during autumn and winter. Studies have shown that the warm and cold phases of ENSO have different impacts on the pattern of weather regimes during the colder season in Europe [32]. Strong El Niño events were related to a higher sea level pressure, a lower T, and dry air in the Baltic countries [32, 41, 42]. According to our data, during the second half of autumn, the NINO3.4 > 1.14 period was characterised by a lower mean AP, more precipitation and RH, and a very significantly lower diurnal temperature range (DTR) (by 1.3 °C, *p* < 0.001). In winter, the NINO3.4 > 1.14 period was characterised by a lower T, WS, and CC and a higher AP and DTR. These weather patterns may be associated with a lower risk of SAH. Some studies have shown that a higher DTR is significantly associated with higher mortality, and this effect was stronger during autumn [43, 44]. Therefore, a lower DTR during autumn may have a positive effect on human health.

We found a positive association between the EA/WRI and HS and an increase in the risk of IS during a strong negative EA/WR phase. These effects were similar during both the colder and the warmer periods, and the additional inclusion of the NAOI in the model did not reduce the significance of the EA/WRI. In the studied region, in winter, the positive EA/WR produced cold advection from the north and was characterised by a lower air temperature, a lower precipitation level, and stronger atmospheric circulation [45]. In the southeastern region of the Baltic Sea, the EA/WRI is negatively correlated with air temperature in spring [46], lake water temperature in spring-autumn [47], and precipitation amount in summer [45]. According to our data, during the positive EA/WR phase, a higher mean WS and AP in winter and a lower mean T and a higher mean AP both in spring and summer were observed. Apart from this, a higher variation was found in the daily change in AP both in winter and spring. Thus, a higher EA/WRI was related to a stronger variation in AP, colder air flow in winter, and colder air in other seasons. The complexity of these weather patterns may be associated with a higher risk of HS.

For the first time, negative correlations between IS and AO and between IS and NAO only during November–March were found. According to the results of studies by other authors, AO was associated not only with tropospheric variability but also with stratospheric variability and changes in weather patterns in Lithuania and nearby regions [48, 49]. During January–March, a positive AO brings a higher surface T and a lower precipitation in middle-latitude regions [50]. In the region of the Baltic Sea, a positive correlation between T and AO was observed during January–March [51], March–May [52], July and October [46], and September–March [53]. According to our data, the AOI was positively correlated with T in all seasons, excluding summer, and negatively correlated with RH, excluding winter. It is possible that this complex of weather patterns (a higher T and a lower RH during the equinox, a lower RH in summer, and warmer winters) related to days of a higher AOI had a protective effect against the risk of IS.

The SCAI was positively correlated with T in summer and negatively correlated with T and positively correlated with AP over the region of the Baltic Sea in winter [26, 31, 46]; the same associations were found in our study. The positive phase of SCA indicates more likely anticyclonic conditions and a lower level of atmospheric circulation over the Baltic Sea region during autumn-spring [26], and the anticyclonic conditions over Scandinavia substantially suppress westerly zonal airflow in summer [54]. It is possible that cold outbreaks during the colder period and the atmospheric variations related to a stronger positive SCA are associated with the risk of IS.

During the colder period, a positive NAO had a protective effect against IS, but a negative NAO had a protective effect against HS. During wintertime, in the Baltic Sea region, a positive NAOI was associated with a higher T and with altered weather: a higher WS, a lower AP, and a northeastward shift in the Atlantic storm activity with enhanced activity from Newfoundland into Northern Europe [25]. As a positive NAO during the winter was associated with more changing weather, a positive NAO was risky for HS, whereas a change in T was more relevant for IS. Studies in Northern and Middle Europe have shown a higher risk of HS associated with changing weather but not with T [19, 20, 22, 38]. A study conducted in the UK showed a significant impact of changes in T only on IS, whereas changes in AP had a significant impact only on the risk of HS [55].

According to the literature, both positive and negative NAO phases are associated with worse health outcomes [56]. found a positive association between the daily AOI with a lag of 3 days and the incidence of and mortality from acute myocardial infarction in Northern Sweden. An inverse association between the climate index (which represents winters with a strong negative phase of the NAO) and the level of mortality from ischaemic heart disease was found in England [57]. In addition, a negative association between the NAO index and systolic and diastolic blood pressure during spring-autumn was found [58].

The pathophysiological mechanisms underlying the correlation between stroke and weather conditions have been discussed. Factors that increase the risk of stroke include high blood pressure, some diseases, and the lack of regular exercise. Blood pressure is influenced by cold, stress and physical activity [58]. Donkelaar et al. [22] hypothesised that AP changes trigger the inflammation process in the aneurysm wall. Variations in AP may influence vessel walls and endothelial function by endogenous inflammatory mechanisms [21]. Studies on thrombosis in air travel suggest that prothrombin fragments and the thrombin-antithrombin complex are activated in hypobaric conditions [59, 60], which could be another clue to the underlying mechanism.

Studies on the associations between physical activity in the elderly and weather conditions in Europe showed that physical activity decreased with increasing WS, precipitation, humidity, and a shorter duration of sunshine [61, 62]. These weather conditions are associated with a negative AO and NAO excluding winter months; therefore, it can be assumed that negative AOs are associated with fewer physical activity opportunities for the elderly, who are likely to be stressed. Thisal activity can explain why a negative AO increased the risk of IS.

The present study has several **strengths** the inclusion of a large number of patients with various types and subtypes of stroke, the long study period, and standardised methods and criteria used for stroke registration. In addition, the present study analyses daily stroke incidence data by stroke subtypes, daily meteorological data, and the variation of these data with respect to the previous day and uses teleconnections such as NAO, AO, EA/WR, SCA, and ENSO. Moreover, the patients that were included coming from a small geographical area (Kaunas) contributes to the homogeneity of weather conditions. In our study, associations between atmospheric circulation patterns such as NAO, AO EA/WR, SCA, and ENSO and the risk of stroke were found for the first time.

The **limitation** is that other potential confounders such as air pollution, influenza epidemics or other respiratory infections were not directly considered in this study. In our region, infections are strongly related to the season with the highest prevalence during winter [63]. In our study, the analyses were controlled for the month and T. Residual confounding by short-term respiratory epidemics remains a possibility. Moreover, we did not consider weather-related physical activity that may have had an impact on individual exposure to outdoor T and humidity.

In our study, the influence of air pollution, which is a known trigger for cardiovascular diseases, was not examined. We did not have air pollution data for the entire study period, but the additional inclusion of the daily concentrations of PM_10_, NO_2_, or O_3_ did not change the association between the risk of strokes and teleconnection indices. Based on the results published by other authors [64], we can assume that the short-term effect of PM_10_, NO_2_, or O_3_ on the risk of stroke was not significant enough to affect the results of our study. First, the level of air pollution in Kaunas is not high. Second, our stroke patients were relatively young (< 65 years of age), whereas in other studies presenting a positive association between air pollution and stroke (except for those performed in subtropics or in regions with high levels of pollutants), the mean age of the patients was over 70 years [64]. Third, we did not evaluate other comorbidities such as acute myocardial infarction, ischaemic heart disease, arterial hypertension, heart failure or other risk factors such as atrial fibrillation, diabetes, dyslipidaemia, or renal or malignant diseases, which may also be associated with a higher risk of ischaemic and haemorrhagic stroke. Fourth, harmful lifestyle factors such as alcohol consumption or smoking, which increase the risk of haemorrhagic stroke, cannot be ruled out, either.

## Conclusion

A protective effect of a stronger El Niño on SAH was found. The risk of HS was associated with an increase in daily AP, a higher CC, and a higher EA/WR index. The risk of IS was negatively associated with the AO index, and a negative impact of a strong positive SCA on the risk of IS was found. During the colder period, a positive NAO had a protective effect against IS, but, vice versa, a negative NAO had a protective effect against HS. The results of our study provide new evidence that the NAO, AO, SCA, EA/WR, and ENSO patterns may affect the risk of stroke. The impact of these teleconnections on various types of stroke is not identical. As U-shaped or J-shaped relationships between daily changes in weather variables and stroke events were found, we recommend using the weather variables as categorical factors in the regression models. Emergency services should be aware that specific weather conditions are more likely to prompt calls for more severe strokes.

## Data Availability

Data that were used in this article have not open access. These data can be accessible to university scientific staff with the permission of the head of the Laboratory of Population Studies.

## Abbreviations

IS: Ischaemic stroke
SAHs: Subarachnoid haemorrhages
ICHs: Intracerebral haemorrhages
EA/WR: East Atlantic/West Russia pattern
HS: Haemorrhagic stroke
AO: Arctic Oscillation
NAO: North Atlantic Oscillation
T: Temperature
AP: Atmospheric pressure
SCA: Scandinavian pattern
ENSO: El Niño-Southern Oscillation
RH: Relative humidity
WS: Wind speed
WHO: World Health Organisation
CT: Computer tomography
AOI: Arctic Oscillation index
NAOI: North Atlantic Oscillation index
EA/WRI: West Russia index
SCAI: Scandinavian pattern index
CC: Cloud cover
CRT: Classification and regression tree
